# Red cell distribution width: a novel and accessible predictor for immune checkpoint inhibitor-associated myocarditis—a retrospective cohort study

**DOI:** 10.3389/fcvm.2026.1737093

**Published:** 2026-06-26

**Authors:** Shili Zhong, Hao Zhang, Meicong Zhao, Zhu Yuan, Zhen Wang, Zhengbin Wu

**Affiliations:** Department of Critical Care Medicine, Daping Hospital, Army Medical University, Chongqing, China

**Keywords:** immune checkpoint inhibitor, myocarditis, red cell distribution width, troponin, vira

## Abstract

**Background:**

Immune checkpoint inhibitor (ICI)-associated myocarditis is a rare but potentially fatal immune-related adverse event characterized by high mortality and the absence of specific diagnostic biomarkers. Red blood cell distribution width (RDW), a routine hematologic parameter reflecting erythrocyte volume heterogeneity, has been linked to cardiovascular and inflammatory disease outcomes. However, its diagnostic relevance in ICI-associated myocarditis remains unclear.

**Methods:**

A single-center retrospective study was conducted from January 2020 to January 2024, including 51 patients with PD-1 inhibitor-associated myocarditis (case group), 58 patients with viral myocarditis (Control 1), and 50 anti-PD-1-treated patients without myocarditis (Control 2). Clinical and laboratory data, including RDW and troponin T (TnT), were analyzed. Statistical tests included the Kolmogorov–Smirnov test, one-way ANOVA, logistic regression, and receiver operating characteristic (ROC) curve analysis.

**Results:**

The mean onset time after PD-1 inhibition therapy was 153.5 ± 184.5 days, and the mean hospitalization period was 13.6 ± 17.5 days, with a survival rate of 33%. RDW was significantly elevated in the case group compared with both control groups (*P* < 0.05). Multivariate logistic regression identified RDW (OR = 3.78) and ln(TnT) (OR = 12.12) as independent factors associated with ICI-associated myocarditis (*P* < 0.05). ROC analysis demonstrated that RDW achieved an area under the curve (AUC) of 0.8882 (cut-off = 14.65; sensitivity 85.6%, specificity 90.5%), which was comparable to TnT (AUC = 0.9102).

**Conclusion:**

RDW, an easily obtainable routine laboratory parameter, is independently associated with ICI-associated myocarditis and shows high and comparable diagnostic accuracy relative to TnT. RDW may serve as a practical, noninvasive, complementary auxiliary indicator for early screening and risk stratification of ICI-associated myocarditis.

## 1 Introduction

In recent years, rapid progress in oncology has driven the extensive clinical application of immune checkpoint inhibitors (ICIs), leading to a global rise in immune-related toxicities. Among these adverse events, ICI-induced myocarditis exhibits the highest mortality rate and represents a major cause of short-term mortality in affected patients (1). The growing number of reported cases of checkpoint inhibitor-associated myocarditis reflects increasing clinical concern about its severity and management (2). Evidence from recent literature and guidelines indicates that the mortality rate of ICI-associated myocarditis remains high and that reliable diagnostic indicators are still lacking (3). Red blood cell distribution width (RDW) is a routinely assessed hematological parameter that reflects the variation in red blood cell size and volume, readily obtainable from standard complete blood count tests (4). Recent studies have demonstrated that RDW is associated with the clinical outcomes of cardiovascular diseases, sepsis, and several other pathological conditions (5–8). Abnormal erythrocyte heterogeneity often results from delayed clearance of red blood cells, increased erythrocyte fragmentation, and impaired maturation under pathological conditions (9).

The association between RDW and inflammation was further discussed in the review by Goyal et al. (10). ICI-associated myocarditis is a rare but life-threatening cardiac adverse event, with its pathogenesis involving systemic changes, local myocardial disruption, and estrogen regulation: (1). Systemically, abnormal circulating immune cells disrupt immune homeostasis; shared antigens among tumor, cardiac, and skeletal muscle cells drive clonal T-cell expansion (which attacks the myocardium by breaking immune tolerance); and thymic dysfunction indirectly promotes abnormal immune responses (11–14). (2). Locally, immune cells (e.g., T cells, macrophages) infiltrate myocardial tissue; crosstalk between these cells amplifies inflammation; and T cells specifically recognize cardiac antigens to damage cardiomyocytes (15–18). Additionally, estrogen level fluctuations may weaken myocardial protective signaling, indirectly increasing disease risk or severity. However, the potential role of RDW in ICI-related myocarditis has not yet been elucidated.

Previous research by our group identified a correlation between elevated RDW and poor prognosis in hemophagocytic syndrome. Based on these findings, the present study aims to investigate the role of RDW in ICI-associated myocarditis and its possible involvement in disease progression and prognosis.

## 2 Methods

### 2.1 Ethics committee approval

The study was approved by the Ethics Committee of Daping Hospital, Army Medical University [Approval No.2024(125), April 25, 2024] and conducted in accordance with the Declaration of Helsinki (1975, revised in 2013; http://www.wma.net/en/20activities/10ethics/10helsinki/).

### 2.2 Patients

**Figure 1:**
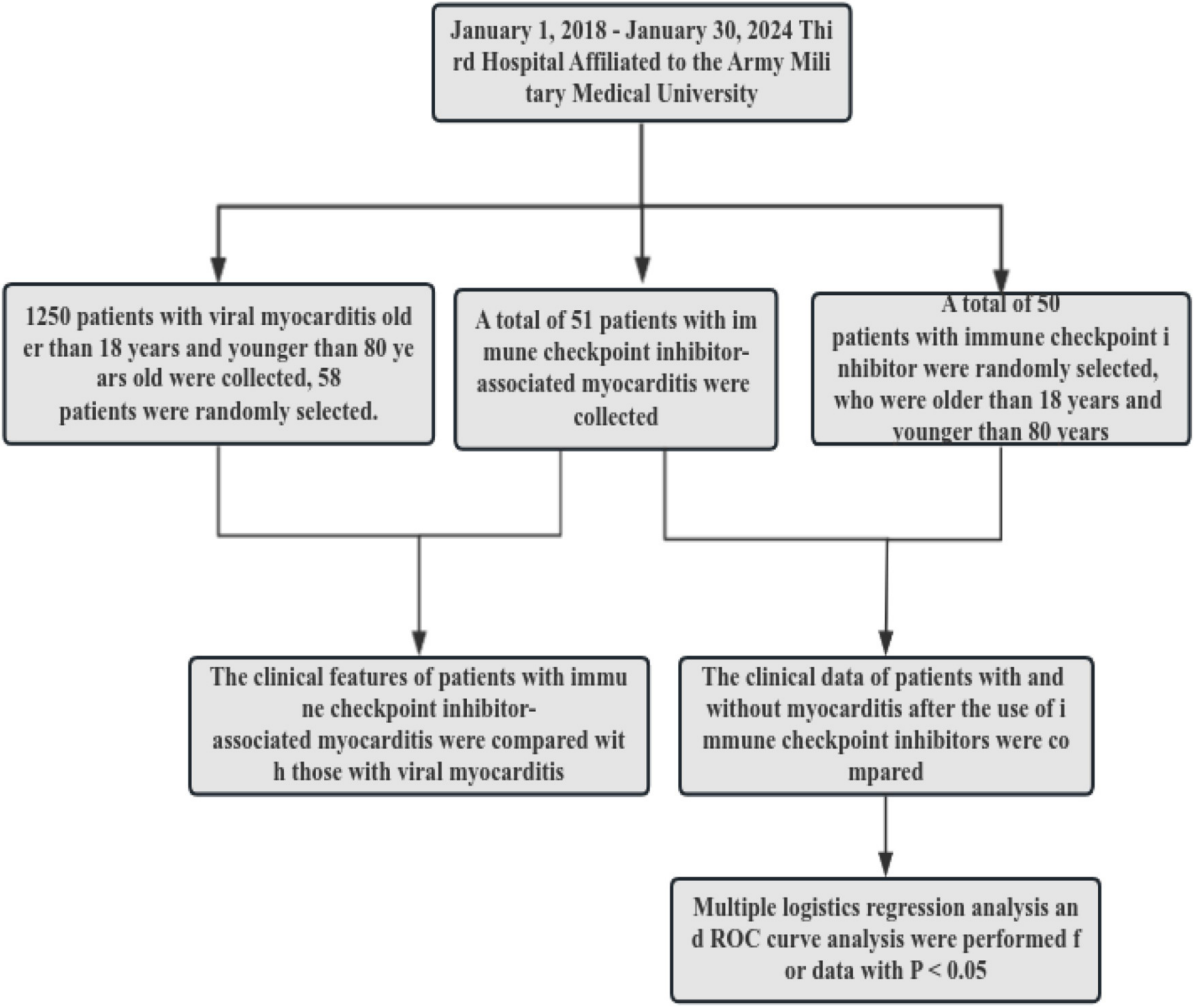
Flow chart for study selection process.

A single-center retrospective study was conducted between January 1, 2020, and January 30, 2024. Fifty-one patients diagnosed with ICI-associated myocarditis who had received PD-1 inhibitors were included as the case group. A total of 1,250 patients with viral myocarditis who met the inclusion criteria were screened, and 58 were randomly selected as the control group using the random number method. In addition, 3,570 cancer patients eligible for ICI therapy were reviewed, and 50 individuals receiving PD-1 inhibitors were randomly selected as a secondary control group (Figure 1). For Control 2 (anti-PD-1-treated non-myocarditis patients), 3,570 eligible patients were first screened to exclude those with a history of cardiovascular disease or anemia (added as exclusion criteria, see Section 2.3). A total of 1,200 patients remained, and 50 were randomly selected using a computer-generated random number table (random number range: 1–1,200, selected numbers corresponding to patient IDs were included) to ensure matching with the case group in terms of cancer type and anti-PD-1 treatment duration.

### 2.3 Diagnostic criteria for ICI-associated myocarditis

Diagnosis of ICI-associated myocarditis is challenging, and there is currently no unified standard. In clinical practice, a comprehensive judgment is usually made based on clinical manifestations, laboratory tests, imaging features, and histological evidence. In 2019, the American Heart Association (AHA) proposed diagnostic criteria for ICI-associated myocarditis, including: (1) Acute or subacute myocardial injury occurring during or after ICI treatment; (2) Exclusion of other causes (such as ischemic heart disease, infection, etc.); (3) At least one of the following: (a). Elevated myocardial enzymes; (b). Abnormal electrocardiogram; (c). Abnormal cardiac imaging; (d). Myocarditis confirmed by myocardial biopsy (3). This standard provides a framework for clinical diagnosis but still has certain subjectivity in practical application. Myocardial biopsy is the gold standard for diagnosing ICI-associated myocarditis, with typical manifestations of infiltration of CD3+, CD4+, CD8+ T cells and CD68+ macrophages in myocardial tissue (19). However, in actual clinical practice, most patients with underlying tumors cannot tolerate myocardial biopsy. In this study, 7 patients admitted to the ICU and receiving tracheal intubation were diagnosed according to the diagnostic criteria for ICI-associated myocarditis proposed by the AHA in 2019 (3), while the remaining 44 patients were diagnosed by myocardial radionuclide imaging.

### 2.4 Exclusion criteria

(1) Severe anemia (hemoglobin < 80 g/L) or hematopoietic system diseases (e.g., leukemia, myelodysplastic syndrome); (2) Acute infection (body temperature > 38.5°C, white blood cell count > 12 × 10^9^/L) within 72 h of blood sampling; (3) Severe liver or kidney dysfunction (alanine transaminase > 3 ×upper limit of normal, estimated glomerular filtration rate < 30 mL/min/1.73 m^2^); (4) Recent blood transfusion (within 2 weeks) or iron supplementation therapy, which may affect RDW levels.

### 2.5 Parameter measurement

Univariate analysis of variance (ANOVA) was applied to compare non-measurement parameters among the three groups. The analyzed parameters included white blood cell count, hemoglobin, RDW, platelet distribution width, neutrophil percentage, lymphocyte percentage, troponin, creatine phosphokinase isoenzyme, B-type natriuretic peptide (BNP), and myoglobin. All laboratory parameters were collected within 24 h of ICU admission or at the time of myocarditis diagnosis.

### 2.6 Statistical analysis

All statistical analyses were performed using SPSS software (IBM Corp, College Station, Texas, United States). The Kolmogorov–Smirnov test was used to assess the normality of data distribution. Continuous variables following a normal distribution were expressed as mean ± standard deviation, whereas non-normally distributed data were presented as median (P25, P75). Categorical variables were expressed as percentages (%). The chi-square test was employed for categorical comparisons, and one-way ANOVA was used for group comparisons, with a significance threshold of *P* < 0.05. Homogeneity of variance was verified using the Levene test. Variables that reached statistical significance were included as independent predictors, while myocarditis occurrence served as the dependent outcome in both binary and multivariate logistic regression analyses (*P* < 0.05 considered significant). For the multivariate logistic regression analysis, variables including red blood cell distribution width (RDW), troponin T (TnT), creatine kinase-MB (CK-MB), myoglobin (Mb), brain natriuretic peptide (BNP), and confounding variables (age, anemia, nutritional status, tumor progression) were incorporated. Collinearity among these variables was verified using the Variance Inflation Factor (VIF), with a threshold of VIF < 5 indicating no significant collinearity. In addition, a Cox proportional hazards model was developed to determine prognostic factors. Receiver operating characteristic (ROC) curves were plotted to evaluate the predictive ability of RDW, platelet count, and fibrinogen (FIB), with the area under the curve (AUC) calculated to assess diagnostic accuracy.

## 3 Results

### 3.1 Basic clinical statistics of 51 patients with ICIs

Following PD-1 inhibitor administration, the mean time to onset of myocarditis was 153.5 ± 184.5 days, and the mean duration of hospitalization was 13.6 ± 17.5 days. Approximately 11.8% of patients required transfer to the intensive care unit for advanced management, and the overall survival rate was 33%. One patient diagnosed with ICI-associated myocarditis complicated by myasthenia gravis achieved successful ventilator weaning and tracheostomy closure after 103 days of hospitalization.

### 3.2 Distribution of tumor sites and proportions

As shown in the Table, the distribution of tumor sites among the included patients was as follows: lung cancer accounted for the largest proportion (17 cases, 34%), followed by intestinal cancer (10 cases, 20%), liver cancer (6 cases, 12%), throat cancer and hematological tumors (3 cases each, 6% each), esophageal cancer (5 cases, 10%), nasopharyngeal cancer (2 cases, 4%), and breast cancer, bladder cancer, cervical cancer, and urethral cancer (1 case each, 2% each) (Table 1).

**Table 1:** Distribution of tumor sites and proportions.

Tumor site	Number of examples (n)	Percentage (%)
Throat	3	6%
Nasopharynx	2	4%
Esophagus	5	10%
Breast	1	2%
Lung	17	34%
Intestine	10	20%
Liver	6	12%
Bladder	1	2%
Cervix	1	2%
Blood	3	6%
Urethra	1	2%

### 3.3 Distribution and proportion of immune checkpoint inhibitor (ICI) treatments

As shown in the Table, the distribution and proportion of immune checkpoint inhibitor (ICI) treatments were as follows: Tislelizumab was the most commonly used agent (25 cases, 50%), followed by Camrelizumab (13 cases, 26%). Bevacizumab and Sintilimab were each used in 4 cases (8% each). Pembrolizumab, Pareliprolimumab, Tislelizumab plus Cetuximab, and Tislelizumab plus Bevacizumab were each used in 1 case (2% each) (Table 2).

**Table 2:** Distribution and proportion of immune checkpoint inhibitor (ICI) treatments.

The immune checkpoint inhibitor (ICI) treatment	Number of examples (n)	Percentage (%)
Bevacizumab	4	8%
Camrelizumab	13	26%
Pembrolizumab	1	2%
Pareliprolimumab	1	2%
Tislelizumab	25	50%
Tislelizumab + Cetuximab	1	2%
Tislelizumab + Bevacizumab	1	2%
Sintilimab	4	8%

### 3.4 Comparison of clinical data and laboratory records

(1) A comparison was conducted between the case group (patients with ICI-associated myocarditis, *n* = 51) and control group 1 (patients with viral myocarditis, *n* = 58). Statistically significant differences were observed in age, length of ICU stay, white blood cell count, hemoglobin, RDW, platelet distribution width, lymphocyte percentage, troponin (TnT), myoglobin, history of heart disease, use of corticosteroid therapy, and ventricular enlargement (all *P* < 0.05) (Table 3). Patients in the case group were older than those in the control group. Although total hospitalization duration did not differ significantly, the ICU stay was markedly prolonged in the case group. White blood cell count, platelet distribution width, lymphocyte percentage, TnT, and BNP levels were lower in the case group, whereas RDW was higher. The incidence of preexisting heart disease in the case group (11%) exceeded that in the control group (5.1%), and corticosteroid therapy was used more frequently in the case group (51%) than in the control group (25%). Additionally, ventricular dilation occurred more frequently among patients with ICI-associated myocarditis (39.2%).
(2) The case group (patients with ICI-associated myocarditis, *n* = 51) was also compared with control group 2 (patients who received ICIs but did not develop myocarditis, *n* = 50). Significant differences were identified in ICU stay, RDW, platelet distribution width, neutrophil percentage, lymphocyte percentage, TnT, creatine kinase isoenzyme, BNP, myoglobin, corticosteroid therapy, arrhythmia, tricuspid regurgitation, ventricular wall thickening, atrial enlargement, and ventricular enlargement (all *P* < 0.05) (Table 3). The duration of ICU stay differed significantly between groups, as no patients in the control group 2 required ICU admission. RDW, neutrophil percentage, corticosteroid therapy rate, incidence of arrhythmia, and prevalence of tricuspid regurgitation, ventricular wall thickening, atrial enlargement, and ventricular enlargement were all higher in the case group than in control group 2. Although platelet distribution width varied between groups, the difference was not pronounced. The lymphocyte percentage was markedly lower in the case group relative to the control group (*P* = 0.002).

**Table 3:** Clinical and laboratory data comparison among the three groups.

Characters	Immune checkpoint inhibitor-associated myocarditis (*n* = 51)	Common viral myocarditis (*n* = 58)	Not have myocarditis using immune checkpoint inhibitors (*n* = 50)	*P*_1_ value (*P* < 0.05)	*P*_2_ value (*P* < 0.05)	F
Sex (woman/total, %)	26 (51.4%)	32 (55.6%)	24 (48.6%)	0.843	1	
Age (Mean ± SD)	63.08 ± 9.37	36.97 ± 18.49	58.98 ± 13.32	0	0.2	
Length of stay (Mean ± SD)	13.27 ± 14.57	9.79 ± 8.77	9.26 ± 6.68	0.710	0.456	
Length of stay in the ICU (Mean ± SD)	5.17 ± 9.38	3.24 ± 11.44	0	0	0.004	
Intubate (%)	(11.8%)	(15.52%)	(2%)	0.405	0.055	
White blood cell (10^9^/L), Mean (Min, Max)	6.46 (4.96, 8.88)	7.71 (5.75, 11.93)	6.07 (4.73, 7.63)	0.010	0.195	10.99
Hemoglobin (Mean ± SD)	128.38 ± 22.06	139.50 ± 21.96	120.53 ± 22.68	0	0.062	10.51
Red blood cell distribution width, Mean (Min, Max)	15.8 (14.5, 17.5)	12.9 (12.4, 13.5)	12.8 (12.0, 13.9)	0	0	56.31
Platelet distribution width, Mean (Min, Max)	12.8 (10.6, 14.3)	15.6 (13.68, 16.33)	12.36 ± 2.44	0	0.043	14.40
Neutrophil percentage (Mean ± SD, %)	71.42 ± 12.80	68.56 ± 15.57	64.62 ± 10.70	0.618	0.019	3.04
Lymphocyte percentage (%), Mean (Min, Max)	16.7 (10.4, 23.8)	24.15 (11.73, 34.01)	24.02 ± 8.01	0.031	0.002	4.73
Troponin (µg/L), Mean (Min, Max)	0.04 (0.02, 0.07)	0.07 (0.03, 0.61)	0.009 ± 0.01	0.009	0	6.89
Creatine phosphokinase isoenzyme(µg/L), Mean (Min, Max)	14.38 (2.58, 45)	13.69 (1.93, 46.5)	5.00 ± 7.14	0.490	0	7.32
BNP (pg/mL), Mean (Min, Max)	99 (32,229.5)	510 (108.75, 1328.75	61.29 ± 40.95	0	0.008	11.96
Myoglobin (µg/L), Mean (Min, Max)	117 (46.5, 720)	53.75 (21,240.55)	28.45 ± 15.08	0.046	0	7.32
History of heart disease (%)	11 (22%)	3 (5.1%)	23.5%	0.005	0.855	
Hormone therapy (%)	26 (51%)	15 (25%)	0%	0.004	0	
Arrhythmology (%)	31 (60.8%)	29 (49.2%)	10%	1	0	
Aortic regurgitation (%)	18 (35.3%)	20 (35.2%)	26%	1	0.478	
Mitral regurgitation (%)	17 (33.3%)	28 (32.8%)	8%	0.528	0.242	
Tricuspid regurgitation (%)	22 (42%)	17 (28.8%)	15.7%	0.170	0.004	
Ventricular septum thickened (%)	10 (19.6%)	9 (16%)	16%	0.346	0.018	
Atrial enlargement (%)	20 (39.2%)	19 (32.2%)	2%	0.428	0	
Ventricular enlargement (%)	20 (39.2%)	16 (27.1%)	2%	0.001	0	

*P*1, comparison between the ICI-associated myocarditis group and the viral myocarditis group; *P*2, comparison between the ICI-associated myocarditis group and the anti-PD-1-treated non-myocarditis group.

### 3.5 RDW and TnT are independent risk factors for ICI-associated myocarditis

**Figure 2:**
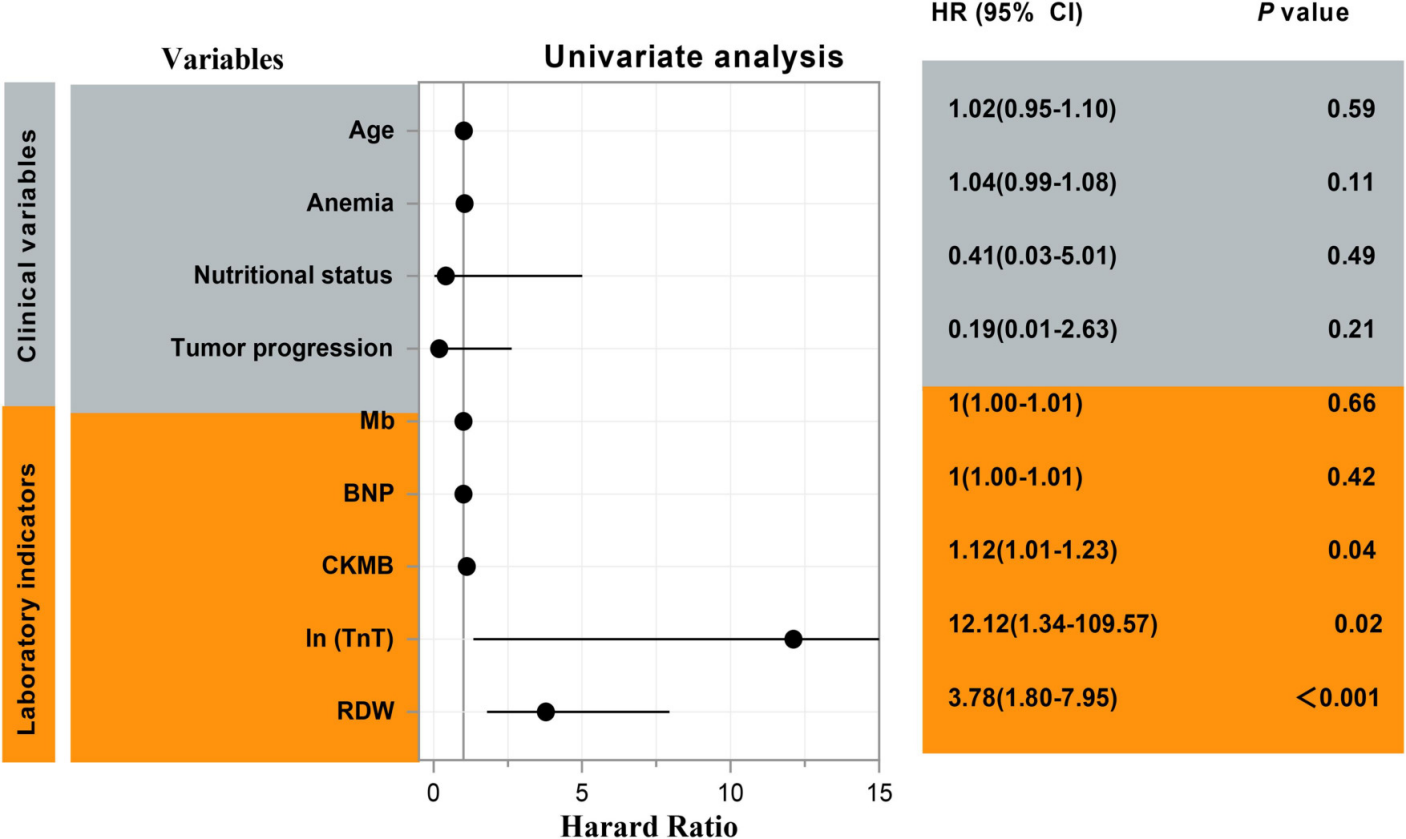
Multifactorial analysis of myocarditis associated with immune checkpoint inhibitors.

Multivariate logistic regression analysis showed that red blood cell distribution width (RDW), creatine kinase-MB (CK-MB), and troponin T (TnT) were significantly associated with ICI-related myocarditis (all *P*-values < 0.05). The odds ratio (OR) of RDW was 3.78 (95% CI: 1.80–7.95, *P* < 0.001), and OR of CK-MB was 1.12 (95% CI: 1.01–1.23, *P* = 0.04). After natural logarithm transformation, ln(TnT) was an independent risk factor (OR = 12.12, 95% CI: 1.34–109.57, *P* = 0.02). All OR values, 95% CIs, and *P*-values were re-checked and confirmed to be statistically consistent (Figure 2).

### 3.6 AUC analysis of RDW and TnT for predicting ICI-associated myocarditis

**Figure 3:**
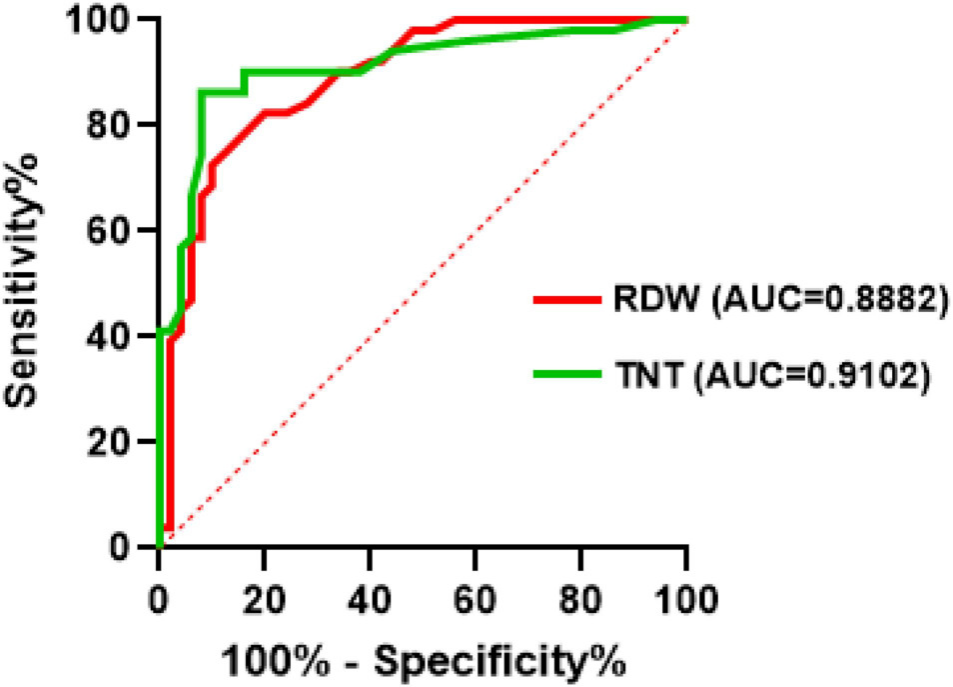
ROC curves of RDW and TnT for predicting ICI-associated myocarditis.

ROC curves were constructed to evaluate the diagnostic performance of RDW and TnT. The AUC of RDW was 0.8882, which was similar and not superior to that of TnT (AUC = 0.9102). Both parameters showed strong diagnostic capacity for ICI-associated myocarditis (Figure 3).

### 3.7 RDW and TnT exhibit high diagnostic performance for ICI-associated myocarditis

The optimal cut-off value for RDW was 14.65%, with a sensitivity of 85.6% and specificity of 90.5%. TnT showed a sensitivity of 86.3% and specificity of 91.8%. These results confirm that RDW provides comparable diagnostic accuracy and may be used as a complementary screening tool rather than a replacement for troponin.

## 4 Discussion

The clinical application of ICIs has revolutionized cancer treatment, offering new therapeutic options for patients with advanced malignancies. However, this progress has been accompanied by the emergence of immune-related adverse events (irAEs), among which ICI-induced myocarditis is a rare but life-threatening complication. Reported incidence rates of ICI-associated myocarditis range from 0.06% to 1%, with mortality rates reaching up to 50% once the condition develops (1, 20–24). Histopathologically, ICI-associated myocarditis is characterized by myofiber necrosis and atrophy, accompanied by infiltration of lymphocytes (predominantly CD8^+^ T cells) and macrophages (19, 25). In a study by Champion et al. (26), 10 patients with ICI-associated myocarditis were classified into high-grade (≥50 CD3^+^ cells per high-power field) and low-grade (<50 CD3^+^ cells per high-power field) subgroups based on inflammatory infiltrate density. Patients in the high-grade subgroup had higher densities of CD3^+^, CD8^+^ T cells, and CD68^+^ macrophages, along with shorter time to onset, more severe clinical manifestations, and lower survival rates. In clinical practice, analyzing peripheral blood lymphocyte subsets can help quantify the proportions of CD3^+^, CD4^+^, and CD8^+^ T cells, aiding in the evaluation of disease severity in ICI-induced myocarditis (27, 36).

In the present study, the mortality rate of ICI-induced myocarditis was 67% (including in-hospital deaths and patients who discontinued treatment). The early stages of ICI-induced myocarditis are often asymptomatic or present with nonspecific symptoms—most patients initially experience tachycardia, chest tightness, or ventricular premature beats. The lack of routine cardiovascular monitoring during ICI therapy contributes to the underestimation of both the incidence and severity of this complication (20). Currently, there are no specific biomarkers or standardized scoring systems for definitive diagnosis (37).

Viral myocarditis was selected as Control 1 in this study to serve as a disease-specific control for myocarditis, aiming to distinguish the unique pathological features and biomarker characteristics of ICI-associated myocarditis from other common etiologies of myocarditis. Viral myocarditis is the most prevalent type of myocarditis in clinical practice, characterized by myocardial inflammation induced by viral infection and subsequent immune response (22, 23, 28), which shares the core clinical feature of myocardial injury with ICI-associated myocarditis. By comparing ICI-associated myocarditis (immune-mediated myocardial injury) with viral myocarditis (infection-induced myocardial injury), we could exclude the interference of “general myocardial injury” on RDW elevation and verify whether RDW elevation is specific to the immune dysregulation induced by ICIs, rather than a nonspecific response to any type of myocarditis. This comparison helps clarify the unique association between RDW and ICI-related immune activation, further supporting the specificity of RDW as a biomarker for ICI-associated myocarditis.

Our analysis revealed distinct clinical features of ICI-associated myocarditis (case group) compared with viral myocarditis (Control 1): (1) Older age (63.08 ± 9.37 vs. 36.97 ± 18.49 years, *P* < 0.001), consistent with the older demographic of cancer patients receiving ICI therapy; (2) Lower BNP levels (99 vs. 510 pg/mL, *P* < 0.001), suggesting less severe heart failure in ICI-associated myocarditis; (3) Higher corticosteroid use rate (51% vs. 25%, *P* = 0.004), reflecting differences in treatment strategies (corticosteroids as first-line therapy for ICI myocarditis vs. supportive care for viral myocarditis). Compared with anti-PD-1-treated non-myocarditis patients (Control 2), ICI-associated myocarditis patients had significantly higher RDW (15.8 vs. 12.8%, *P* < 0.001) and TnT (0.04 vs. 0.009 μg/L, *P* < 0.001)—highlighting the potential of these biomarkers to distinguish myocarditis from cancer-related systemic changes.

To address the potential impact of these baseline differences on the study results, we incorporated key confounding factors (including age, anemia, nutritional status, and tumor progression) into the multivariate logistic regression analysis. The results confirmed that RDW (OR = 3.78, *P* < 0.05) and ln(TnT) (OR = 12.12, *P* < 0.05) remained independent risk factors for ICI-associated myocarditis after adjusting for these baseline differences. This indicates that the observed association between RDW elevation and ICI-associated myocarditis is not confounded by age or other baseline variables, and the baseline differences do not affect the validity of the study conclusions.

RDW elevation can be influenced by multiple confounding factors, including anemia, acute infection, nutritional deficiencies, hematopoietic system diseases, liver/kidney dysfunction, recent blood transfusion, and iron supplementation therapy—all of which may independently affect red blood cell size heterogeneity and confound the association between RDW and ICI-associated myocarditis. To minimize the impact of these confounders, we implemented a comprehensive control strategy throughout the study: (1) Strict exclusion criteria: We excluded patients with severe anemia (hemoglobin < 80 g/L), hematopoietic system diseases (e.g., leukemia, myelodysplastic syndrome), acute infection (body temperature > 38.5°C℃, white blood cell count > 12 × 10^9^/L within 72 h of blood sampling), severe liver or kidney dysfunction (alanine transaminase > 3 × upper limit of normal, estimated glomerular filtration rate < 30 mL/min/1.73 m^2^), and recent blood transfusion (within 2 weeks) or iron supplementation therapy—all factors known to affect RDW levels. (2) Matching of Control 2: For Control 2 (anti-PD-1-treated non-myocarditis patients), we first screened 3,570 eligible patients to exclude those with a history of cardiovascular disease or anemia (which may affect RDW), then randomly selected 50 patients who were matched with the case group in terms of cancer type and anti-PD-1 treatment duration. This matching reduced the confounding effect of cancer-related factors and ICI treatment duration on RDW levels. (3) Multivariate adjustment: In the logistic regression analysis, we incorporated potential confounders (age, anemia, nutritional status, tumor progression, and other laboratory parameters) into the model. Collinearity among variables was verified using the Variance Inflation Factor (VIF), with VIF < 5 indicating no significant collinearity. The results confirmed that RDW was independently associated with ICI-associated myocarditis after adjusting for these confounders, indicating that the observed RDW elevation is specifically related to ICI-induced immune dysregulation and myocardial injury, rather than other confounding factors.

The 2017 National Consensus on Monitoring and Management of ICI-Related Myocarditis emphasized that early monitoring, timely diagnosis, and stratified intervention can significantly reduce mortality (29). Although endomyocardial biopsy remains the diagnostic gold standard (19), it carries inherent risks such as cardiac perforation and bleeding. For patients with malignancy, invasive biopsy is rarely preferred due to poor tolerance and compromised clinical status. Thus, clinical diagnosis often relies on a combination of ICI treatment history, clinical manifestations, cardiac injury biomarkers, myocardial enzyme levels, echocardiography, and cardiac magnetic resonance imaging (CMRI). However, studies have shown that the diagnostic sensitivity of CMRI for ICI-induced myocarditis is lower than that for viral myocarditis (30, 31). Additionally, CMRI is costly and technically demanding, limiting its use as a routine diagnostic tool.

In our comparative analysis of clinical data from patients with ICI-induced myocarditis, viral myocarditis, and anti-PD-1-treated non-myocarditis patients, both RDW and TnT were strongly associated with ICI-associated myocarditis. RDW levels were markedly elevated in the case group compared with both control groups, and elevated RDW and TnT may serve as valuable auxiliary indicators for the early detection of ICI-induced myocarditis—enabling timely risk stratification and individualized management.

The elevated red blood cell distribution width (RDW) in immune checkpoint inhibitor (ICI)-associated myocarditis is mainly caused by the interaction of immune activation, systemic inflammation, myocardial injury and disrupted erythropoiesis. As a marker of red blood cell size heterogeneity, RDW is a promising biomarker for this disease. ICIs induce excessive immune activation, including cardiac myosin-specific autoimmune T cells, gasdermin-E-mediated pyroptosis and cGAS-STING signaling activation, which drive myocardial inflammation and injury. Mitochondrial damage, complement dysregulation and TLR4 overactivation further amplify inflammation. Three core mechanisms link these processes to elevated RDW: impaired erythropoiesis and increased RBC fragmentation, cytokine-induced iron metabolism disorder, and secondary effects of cancer-related factors. Importantly, regression analyses confirm RDW elevation is independently associated with ICI-associated myocarditis, indicating it specifically reflects immune-mediated myocardial damage rather than general illness, supporting its role as a noninvasive biomarker for disease assessment (20, 21).

In a study by Salem et al. (22), over half of patients (53%) developed cardiac toxicity within 4 weeks of initiating combination ICI therapy, with 17% experiencing toxicity after the first dose and 34% having cardiac events within 4 months of monotherapy. In contrast, the mean time to onset of myocarditis in our study was 153.5 ± 184.5 days, with a mean hospitalization duration of 13.6 ± 17.5 days. According to European guidelines (29), glucocorticoids are recommended as first-line therapy for ICI-induced myocarditis, and treatment should continue until cardiac injury biomarkers return to baseline before tapering or discontinuation. However, in our cohort, only 51% of patients received glucocorticoids, and statistical analysis indicated that both treatment duration and initial dosage were inadequate—factors that may have contributed to prolonged hospitalization and higher mortality. For patients refractory to corticosteroid therapy, intravenous immunoglobulin (IVIG) remains a recommended second-line option (26).

Myocarditis, myositis, and myasthenia gravis are collectively termed the “ICI-related triad”—a rare but severe complication of ICI therapy (27, 28, 32–34, 38). Among the 51 patients with ICI-induced myocarditis in our study, one case of overlap syndrome (myocarditis combined with myasthenia gravis) was identified—an exceptionally rare presentation. This patient experienced recurrent ventricular fibrillation refractory to lidocaine, esmolol, amiodarone, and electrical cardioversion. Administration of 1 g methylprednisolone pulse therapy resulted in marked improvement in arrhythmia. After more than three months of intensive care, the patient was successfully weaned from mechanical ventilation and underwent tracheostomy closure. In a report by Dong et al. (35), among 2,550 patients with urothelial carcinoma who received ICI therapy, 111 developed cardiac adverse events (CAEs)—yielding an incidence of 0.22–10.60%, with grade 3–5 events accounting for 52.3%. The most common CAEs were hypertension (28.8%), arrhythmia (14.4%), and hypotension (6.3%)—emphasizing the need for early blood pressure and cardiac monitoring during ICI therapy.

Troponin T remains the gold-standard biomarker for myocardial injury in routine clinical practice. Our results do not indicate that RDW outperforms TnT; instead, RDW exhibited comparable diagnostic accuracy and may serve as an accessible, complementary auxiliary indicator for early screening of ICI-associated myocarditis. As a routine, low-cost parameter derived from complete blood count, RDW provides added value for risk stratification, particularly in settings where timely troponin measurement is not readily available.

## 5 Limitations

This study has several limitations. First, it was a single-center retrospective study with a relatively small sample size (51 cases), which may limit the generalizability of the findings. Second, due to the retrospective design, some data (e.g., long-term follow-up outcomes) were incomplete. Third, we did not explore the dynamic changes in RDW during ICI therapy, which may provide additional insights into its prognostic value. Future prospective, multicenter studies with larger sample sizes are needed to validate our findings and further investigate the role of RDW in ICI-associated myocarditis.

## Data Availability

The data analyzed in this study is subject to the following licenses/restrictions: Data sets are available from the corresponding author upon request. Requests to access these datasets should be directed to Zhengbin Wu email: wuzb@tmmu.edu.cn.
